# Tuberculosis incidence among household contacts of individuals with symptomatic and asymptomatic tuberculosis: A historical cohort study in the Western Cape province of South Africa

**DOI:** 10.1371/journal.pgph.0006550

**Published:** 2026-10-29

**Authors:** Lauren R. Brown, James A. Seddon, Christopher Kelly, Mariette Smith, Peter Godfrey-Faussett, Rory Dunbar, Andrew Boulle, Georgia A. Roussos, Mareli Claassens, Cari van Schalkwyk

**Affiliations:** 1 South African Centre for Epidemiological Modelling and Analysis (SACEMA), Centre for Epidemic Response and Innovation (CERI), School for Data Science and Computational Thinking, Stellenbosch University, Stellenbosch, South Africa; 2 Department of Paediatrics and Child Health, Desmond Tutu TB Centre, Stellenbosch University, Cape Town, South Africa; 3 Department of Infectious Disease, Imperial College London, London, United Kingdom; 4 Western Cape Department of Health and Wellness, Provincial Health Data Centre, Cape Town, South Africa; 5 Centre for Integrated Data and Epidemiological Research, University of Cape Town, Cape Town, South Africa; 6 Faculty of Infectious and Tropical Diseases, London School of Hygiene & Tropical Medicine, London, United Kingdom; 7 Department of Human, Translational and Biological Medical Sciences, School of Medicine, University of Namibia, Windhoek, Namibia; PLOS: Public Library of Science, UNITED STATES OF AMERICA

## Abstract

Longitudinal, individual-level data describing long-term tuberculosis (TB) risk in high-burden communities are limited. We linked participants from the 2010 Zambia, South Africa Tuberculosis and AIDS Reduction (ZAMSTAR) prevalence survey to routine health records in the Western Cape Provincial Health Data Centre (PHDC) to estimate TB incidence of diagnosis over 11 years (to December 2020) among individuals without prevalent TB at enrolment. Cox proportional hazards models assessed predictors of TB diagnosis, including sex, age, HIV status, and household exposure to symptomatic or asymptomatic TB. Of 30,017 participants enrolled in the ZAMSTAR prevalence survey, 15,887 (52.9%) were linked to the PHDC, including 15,471 without prevalent TB at enrolment. Of the 15,471 individuals, 1,819 (11.8%) developed incident TB over the subsequent 11 years. TB incidence declined from 1,614 per 100,000 person-years in 2011–1,302 per 100,000 person-years in 2019. Over follow-up, individuals who had been exposed to symptomatic TB within their household had the highest cumulative probability of TB diagnosis (13%; 95% CI: 7.7–18%). In a multivariable Cox model adjusted for sex and HIV status, individuals living with a household contact with symptomatic TB had a higher hazard of TB incidence than those from unexposed households (aHR 1.53, 95% CI: 1.09–2.13); the hazard of incident TB among those exposed to asymptomatic TB was not significantly elevated (aHR 1.12, 95% CI: 0.67–1.87). In sensitivity analyses restricting follow-up to two and three years, hazard ratios were higher among individuals living with a symptomatic household contact (two years: 1.82 (95% CI: 0.97–3.41) and three years: 1.73 (95% CI: 0.98–3.10)). This long-term linked cohort demonstrates sustained TB incidence in this high-burden community. Incomplete linkage of individuals to the PHDC highlights the need for improving data collection in research studies and national patient identification systems to support long-term cohort analyses.

## Introduction

Despite considerable progress over recent decades, much remains to be done to accelerate declines in tuberculosis (TB) incidence [1]. A persistent gap between estimated TB incidence and notified individuals continues to undermine control efforts, particularly in high-burden settings such as South Africa [2]. A major contributor to this gap is the large proportion of individuals with undetected or unreported TB who do not seek care, perhaps because they have asymptomatic disease [3]. The 2017–2018 South African national prevalence survey found that more than half of individuals with bacteriologically confirmed TB reported no symptoms, [4] as shown in other settings [5–7]. This highlights the reservoir of disease that is unlikely to be identified through passive or symptom-based case finding.

Traditional symptom-based detection approaches fail to detect individuals with early, mild, or non-specific manifestations of TB [8,9]. The recently proposed International Consensus for Early TB framework refines the conceptualisation of TB disease by distinguishing infectiousness, clinical presentation, and pathological extent [10]. This framework highlights the possibility that individuals without classic symptoms may still have viable or transmissible infection. Despite the growing recognition of the importance of asymptomatic disease, the duration, natural history, and contribution of asymptomatic TB to community transmission remain poorly characterised.

Measuring infectiousness directly is challenging. Methods such as aerosol sampling, [11,12] tuberculin skin tests or interferon-gamma release assays among contacts, [13] and prospective follow-up of household contacts [14] provide indirect evidence of transmission potential but require intensive designs that are difficult to scale and rarely linked to large, representative community surveys. Consequently, most estimates of the natural history and downstream outcomes of asymptomatic TB rely on cross-sectional data with limited follow-up.

The Zambia, South Africa Tuberculosis and AIDS Reduction (ZAMSTAR) study was a cluster-randomised trial which implemented two interventions (community-level enhanced case finding and household HIV/TB care) in selected communities across the two countries. [15, 16] As part of the study, a population-based TB prevalence survey was conducted in 2010, approximately 3.5 – 4.5 years after intervention initiation, among randomly selected adults (≥18 years) in each community. The primary outcome was the prevalence of culture-confirmed pulmonary TB, defined by the isolation of *Mycobacterium tuberculosis* from at least one respiratory sample, regardless of symptom status. In the Western Cape, the survey was conducted across eight communities and collected detailed individual-level data, including sputum culture results, TB symptom screening, HIV status, and household exposure characteristics. While well suited to describing the cross-sectional epidemiology of symptomatic and asymptomatic TB, the study design did not include longitudinal follow-up, limiting inference on disease progression or subsequent TB risk.

In the Western Cape, linkage of ZAMSTAR survey participants to the Provincial Health Data Centre (PHDC) provides a rare opportunity to overcome these limitations. The PHDC integrates person-level data across the province’s public-sector health information systems, from sources as far back as 1994, offering a more complete record of TB diagnoses than TB treatment registers alone [17]. Deterministic linkage of ZAMSTAR participants to the PHDC enables long-term follow-up of individuals identified through the survey, allowing the estimation of TB incidence, detailed characterisation of the natural history and care-engagement patterns of asymptomatic TB, and assessment of key demographic and clinical predictors of subsequent disease.

In this study, we analysed data on ZAMSTAR participants residing in the Western Cape to estimate TB incidence of diagnosis between 2010–2020 among those without prevalent TB during ZAMSTAR, and evaluated predictors of incident TB, including sex, age, HIV status, and household symptom status.

## Materials and methods

### Ethics statement

The ZAMSTAR trial was approved by the ethics committees of the London School of Hygiene and Tropical Medicine, Stellenbosch University, and the University of Zambia. Additional ethics approval and permission for the current analysis were obtained from Stellenbosch University and the Western Cape Provincial Department of Health. De-identified data were obtained on 17 May 2024 and no authors had access to information that could identify individual participants during or after data collection.

### Data sources and linkage

#### The ZAMSTAR prevalence survey.

The ZAMSTAR prevalence survey enrolled 30,017 consenting adults (≥18 years) in the Western Cape arm, which we used for data linkage to Western Cape health service records. The survey collected demographic data, TB results confirmed by culture for *Mycobacterium tuberculosis*, HIV test results (based on serological testing or self-reported status), household details, and structured symptom screening responses. The symptom screen captured cough, fever, night sweats, weight loss, shortness of breath and chest pain (definitions in S1 Table). Participants were classified as: (i) index participants diagnosed with baseline TB; (ii) household contacts of index participants; and (iii) participants from households without baseline TB. As this survey was conducted alongside the ZAMSTAR trial, [15] some participants may have been exposed to trial interventions.

#### The Western Cape provincial health data centre.

The Western Cape Provincial Health Data Centre (PHDC) integrates individual-level clinical information from multiple electronic health systems across the province [17]. Data sources include laboratory, pharmacy, and clinical service records from public health care facilities, as well as programme-specific datasets such as HIV and TB services [17]. TB episodes are based on laboratory confirmation (culture, smear microscopy, or molecular diagnostics such as Xpert MTB/RIF) or a clinical diagnosis with appropriate ICD-10 codes (S1 Table). Similarly, the PHDC captures HIV episodes, including the timing of HIV diagnosis, enabling HIV status to be tracked over the same period as TB. Mortality is captured through in-hospital records and TB programme notifications. However, as the PHDC is not directly linked to the National Department of Home Affairs vital registration system, deaths occurring outside the health system may have been missed. Data were extracted up to 31 December 2020 and included dates of TB diagnosis, TB treatment outcomes, and HIV status. All data were de-identified prior to this analysis.

#### Data linkage and cohort follow-up.

Demographic identifiers (first name, surname, age, sex, address, and date of birth) were used to deterministically match ZAMSTAR trial participants to PHDC records. More information on the linkage algorithm is provided in the S1 Text, summarised from Mutemaringa and colleagues [18]. Where multiple potential matches were identified, individuals were excluded to ensure high specificity. Incident TB episodes were defined as TB diagnoses recorded in the PHDC after the survey enrolment date. Individuals with baseline TB were excluded from incidence analyses.

### Definitions

Index participants were defined as individuals enrolled in the ZAMSTAR survey with microbiologically confirmed TB at baseline. Symptom status was determined using the structured questionnaire administered during the survey [19]. Symptomatic TB was defined as the presence of ≥1 of the following: prolonged cough (>2 weeks), weight loss, fever, night sweats, chest pain, or shortness of breath. Asymptomatic TB was defined as the absence of all six symptoms. Exploratory analyses restricted to the four WHO symptoms, derived in part from the original ZAMSTAR trial, will be presented in the Supplementary Materials [19,20]. Households were categorised by the symptom status of their index participant: symptomatic household, asymptomatic household, or unexposed households (housed no participant with TB during ZAMSTAR). Households with more than one individual with different symptom statuses were excluded from analyses. For the purpose of this study, TB incidence refers to the occurrence of a TB diagnosis identified through the PHDC as opposed to true TB incidence.

### Statistical analysis

Annual TB incidence rates per 100,000 person-years (/100k person-years) were calculated by dividing the number of incident TB episodes by total person-time contributed over the 11-year study period. Each participant contributed person-time from the date of ZAMSTAR survey participation until the earliest of: (i) incident TB diagnosis, (ii) death, or (iii) the study end date (31 December 2020).

Poisson regression was used to estimate 95% confidence intervals (CIs) for incidence rates. Predictors of TB diagnosis were assessed using Cox proportional hazards models. Hazard ratios (HRs) and adjusted hazard ratios (aHRs) with 95% CIs were estimated for sex, age, HIV status, and household type. Age and HIV status were modelled as time-varying covariates to account for changes during the study timeframe, while sex and household type were fixed. Age was not included in the adjusted analysis due to the complexity introduced by multiple age categories, which may lead to model overfitting. The primary exposure of interest was household TB exposure type. Sex and HIV status were included a priori because they are strong predictors of TB incidence, so that household type hazard ratios are estimated conditional on these variables. Male sex has been shown to be associated with higher TB prevalence (pooled male:female prevalence ratio 2.02, 95% CI: 1.71–2.34) [21], and HIV is a well-established risk factor for TB, with average incidence rate ratios >5 across levels of immunodeficiency [22]. We did not assume that either confounds the association between household exposure and TB. Kaplan–Meier curves were used to compare the cumulative probability of TB diagnosis across household categories. To assess whether associations were diminished over longer follow-up, sensitivity analyses were conducted restricting follow-up to two and three years. Individuals were censored at these time points if no TB diagnosis had occurred, and Cox proportional hazards models were re-estimated using the truncated follow-up time.

## Results

The final analytic cohort derived from the Western Cape arm of the ZAMSTAR prevalence survey comprised 15,887 (52.9%) unique individuals after the removal of unmatched and duplicate records, multiple TB episodes, and individuals living in households containing participants with differing TB symptom statuses (Fig 1). There were 15,471 (51.5%) individuals from the ZAMSTAR survey who did not have TB at baseline. Of those 1,819 (11.8%) developed TB between their enrolment in the ZAMSTAR survey and the end of 2020, 360 (2.3%) experienced multiple episodes, and 969 (6.3%) died.

**Fig 1:**
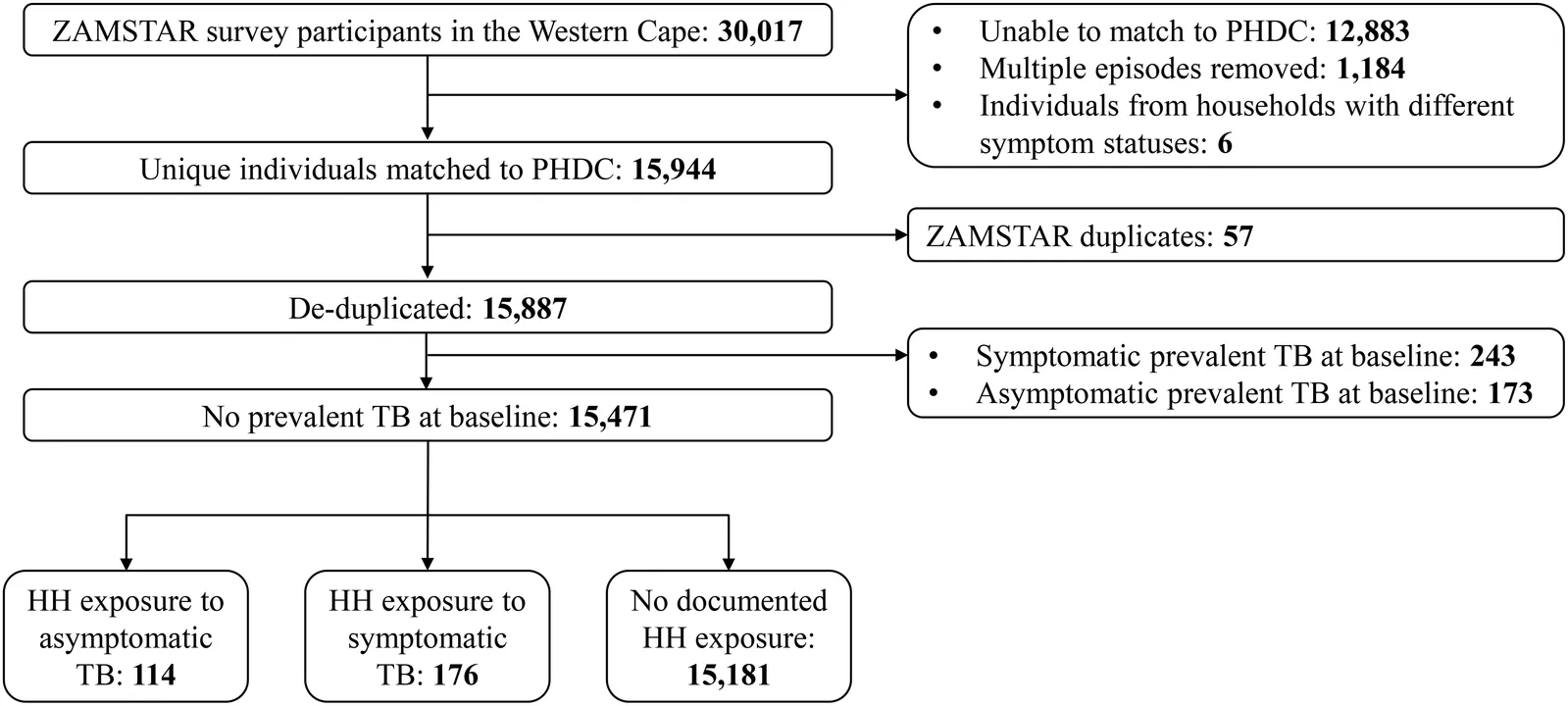
Derivation of the analytic cohort from the ZAMSTAR survey. Of 30,017 adults enrolled in the Western Cape arm of the ZAMSTAR survey in 2010, 15,887 (52.9%) unique individuals were retained for follow-up analyses between 2010 and 2020. ZAMSTAR = Zambia, South Africa Tuberculosis and AIDS Reduction, PHDC = Provincial Health Data Centre, HH = household.

Among individuals without TB at baseline, 176 (1.1%) lived in households with a participant with symptomatic TB, 114 (0.7%) lived in households with a participant with asymptomatic TB, and 15,181 (98.1%) lived in households without a participant with prevalent TB (Table 1). Most participants were aged 18–44 years. Females comprised the majority across all groups (66.5%), consistent with the higher proportion of female participants who consented to sharing their information in the ZAMSTAR prevalence survey and higher linkage of females to the PHDC. Demographic characteristics of the ZAMSTAR cohort and the individuals we were able to match to the PHDC are shown in Supplementary S1 Fig. The majority (58%, 8,907/15,471) of individuals in the cohort matched to the PHDC had a last recorded date of contact with the Western Cape health services after our study end date.

**Table 1:** Descriptive characteristics of the study population without prevalent TB at baseline, N = 15,471.

	Not exposed to household TB	Exposed to symptomatic household TB	Exposed to asymptomatic household TB
Total	15,181 (98.1%)	176 (1.1%)	114 (0.7%)
**Sex**			
Female	10,099 (66.5%)	115 (65.3%)	74 (64.9%)
Male	5,082 (33.5%)	61 (34.7%)	40 (35.1%)
**Age**			
18-24	4,535 (29.9%)	56 (31.8%)	34 (29.8%)
25-34	5,068 (33.4%)	41 (23.3%)	33 (28.9%)
35-44	2,810 (18.5%)	43 (24.4%)	22 (19.3%)
45-54	1,611 (10.6%)	18 (10.2%)	16 (14%)
55-64	784 (5.2%)	11 (6.3%)	7 (6.1%)
65+	373 (2.5%)	7 (4%)	2 (1.8%)
**HIV status**			
Negative	8,437 (55.6%)	99 (56.3%)	52 (45.6%)
Positive	2,302 (15.2%)	28 (15.9%)	15 (13.2%)
Unknown	4,442 (29.3%)	49 (27.8%)	47 (41.2%)

The estimated TB incidence for the full study period was 1,230/100k person-years (95% CI: 1,178 – 1,282). TB incidence was estimated between 1,614/100k person-years (95% CI: 1,426 – 1,826) in 2011 and 1,302/100k person-years (95% CI: 1,134 – 1,494) in 2019 (Fig 2). In 2020, incidence declined by 45.6% to an estimated 708/100k person-years (95% CI: 587.44 – 852.2), likely reflecting individual- and system-level changes due to the COVID-19 pandemic [23,24]. Annual numbers of incident TB, exposure times, and estimated incidence rates are provided in S2 Table.

**Fig 2:**
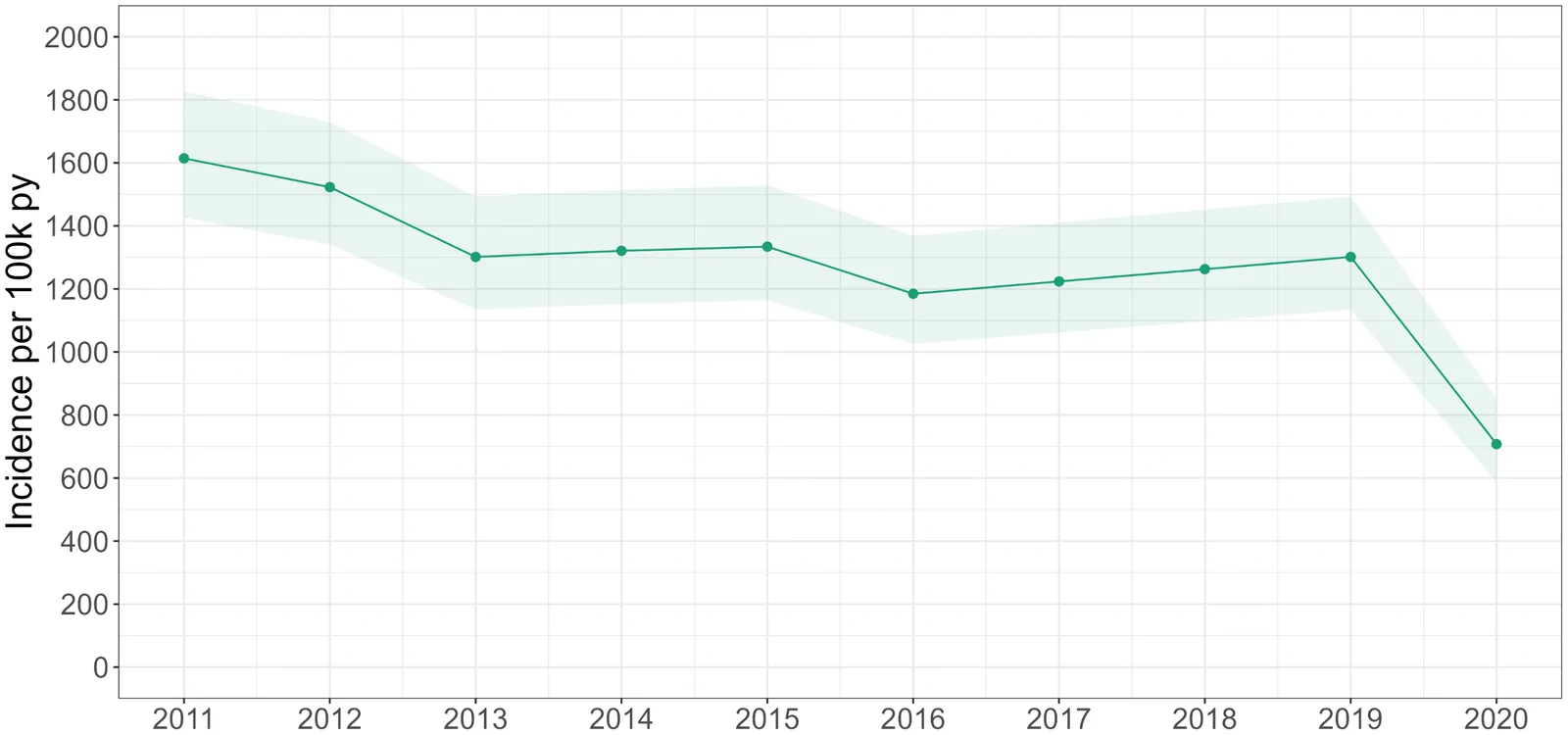
TB incidence per 100,000 person-years between 2011 and 2020 among the 15,471 individuals without prevalent TB at baseline.

The cumulative probability of TB diagnosis differed by household exposure type (Fig 3), with consistently higher probabilities among individuals living in households with a participant with symptomatic TB (though the difference is not significant). Over 11 years of follow-up, individuals living in households with a participant with symptomatic TB had a 13% (95% CI: 7.7–18.1%) probability of TB diagnosis compared with 9.5% (95% CI: 3.7-15%) among those living in households with a participant with asymptomatic TB.

**Fig 3:**
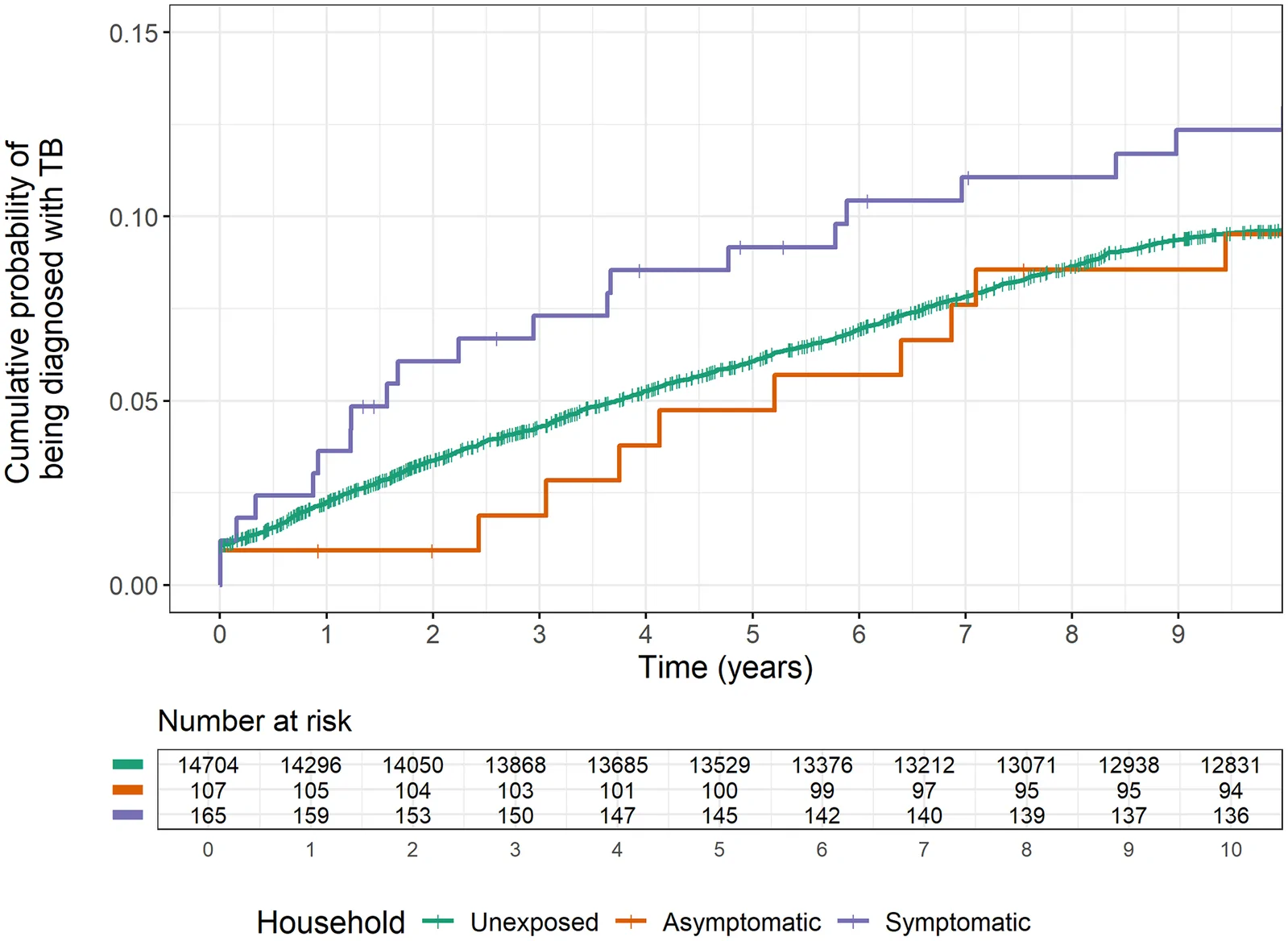
Kaplan-Meier survival curves of the probability of being diagnosed with TB by household type. Green, orange, and purple lines indicate individuals living in households unexposed to TB, or with an individual with asymptomatic or symptomatic TB, respectively. Vertical lines represent censoring from death.

Individuals living in households with a participant with symptomatic TB had a higher risk of TB diagnosis than those from unexposed households (unadjusted HR 1.34, 95% CI: 0.87 – 2.06; aHR 1.53, 95% CI: 1.09 – 2.13) (Table 2), findings using the WHO symptom definition were of similar magnitude (S2 Fig and S3 Table). In analyses restricted to two and three years of follow-up, ten incident TB diagnoses occurred among individuals living with a symptomatic household contact compared with one among those living with an asymptomatic household contact at two years, and 12 versus two at three years; hazard ratios were higher among individuals living with a participant with symptomatic TB, with HRs of 1.82 (95% CI: 0.97 – 3.41) and 1.73 (95% CI: 0.98 – 3.10) at two and three years, respectively. Using the WHO four-symptom definition, the three-year estimate was similar in magnitude (HR 1.78, 95% CI: 1.01 – 3.15; Supplementary S3 Fig and S4 Table)

**Table 2:** Demographics and Cox proportional hazards ratios of TB diagnosis risk by sex, age, HIV status, and household type between 2010 and 2020, N = 15,471.

	Incident TB	HR (95% CI)	aHR (95% CI)
**Household type** ^**‡**^			
Unexposed	1,774	Reference	Reference
Symptomatic	31	1.34 (0.87 – 2.06)	1.53 (1.09 – 2.13)
Asymptomatic	14	0.94 (0.50 – 1.75)	1.12 (0.67 – 1.87)
**Sex**			
Female	1,113	Reference	..
Male	706	1.26 (1.13 – 1.40)	..
Age*			
18-24	472	Reference	..
25-34	646	1.38 (1.18 – 1.63)	..
35-44	393	1.51 (1.27 – 1.80)	..
45-54	213	1.43 (1.18 – 1.74)	..
55-64	67	1.03 (0.79 – 1.33)	..
65+	28	0.89 (0.63 – 1.26)	..
**HIV status**			
Negative	878	Reference	..
Positive	885	2.79 (2.53 – 3.08)	..
Unknown	56	0.13 (0.10 – 0.17)	..

*Age recorded at baseline (during the ZAMSTAR survey)

‡Using the definition of symptomatic TB as at least one of the following: prolonged cough, night sweats, weight loss, fever, chest pain, shortness of breath. Adjusted estimates are not shown for sex, HIV status or age. The adjusted model estimates household type association; sex and HIV status were included as covariates and age was not included.

CI = confidence interval, (a)HR = (adjusted) hazard ratio

## Discussion

Using linkage between data from the 2010 ZAMSTAR survey and 11 years of Western Cape routine records, we estimated that TB incidence in this cohort remained high up to 2019. More than one in ten of the individuals recruited to ZAMSTAR, without prevalent TB at baseline, developed incident TB over the subsequent 11 years. Additionally, we found that living with a household contact with symptomatic TB was associated with an increased hazard of incident TB.

The incidence observed in this cohort is substantially higher than population-level estimates from other subpopulations in the Western Cape. A study of microbiologically confirmed TB in the Western Cape reported 885 per 100,000 population in 2012, [25] while a study of notifications from the Electronic TB Register documented notification rates of 845–713 per 100,000 population in the City of Cape Town between 2010 and 2013, the period and setting from which most Western Cape ZAMSTAR sampling occurred [26]. A longitudinal cohort from a high-burden community in the North West province reported similarly elevated incidence in 2014 (1,300 per 100,000 person-years) [27]. Persistent high incidence in our cohort indicates ongoing transmission and missed detection despite advances in diagnostics and HIV/TB programme scale-up. Subpopulations in the Western Cape arm of the ZAMSTAR survey were, however, targeted because they were located in high TB burden areas identified by experts. Consequently, the estimates presented here may not be directly generalisable, due both to the targeted sampling and to differences in data sources, study design, and follow-up duration. A spatial analysis of household and community contacts in neighbouring, high-TB burden, Uitsig and Ravensmead suburbs showed that high TB incidence in these smaller populations (e.g., households) may be due mostly to social determinants and not just infectious disease epidemiology amongst the populations [28]. The marked drop in incident TB observed in 2020 likely reflects COVID-19-related disruptions to care seeking and service availability; however, the 45.6% decline is substantially larger than that reported for the City of Cape Town (28%) [23] and the Western Cape province overall (22%) [24]. This may reflect characteristics of the study population, such as living in high-burden and socioeconomically vulnerable communities that may have experienced additional barriers to healthcare access during COVID-19 restrictions, though this requires further investigation.

Forty percent of linked ZAMSTAR participants with baseline culture-positive TB were asymptomatic, which highlights the limitations of existing tools and strategies for case detection [9]. Despite this, only 14 of the 114 individuals residing with an asymptomatic index participant were subsequently diagnosed with TB over follow-up. These small numbers limit inference about the incremental risk posed by living with an individual with asymptomatic disease. Nonetheless, these findings suggest that a large reservoir of undetected TB may persist in the community without resulting in elevated household-specific incidence. This has important implications for passive case-finding strategies that rely predominantly on symptom presentation, particularly in high-burden settings where transmission may occur across multiple social and community networks rather than exclusively within households. Larger and more comprehensive studies that ensure rigorous procedures to collect linkage data are required to make definitive conclusions about linkage studies such as this. Separately, ongoing work is examining the health trajectories of the 416 ZAMSTAR index participants with symptomatic or asymptomatic TB who could be linked to the PHDC, to better characterise their disease progression, including retreatment, recurrence, and mortality, over the same 11-year period.

Individuals living with a household contact with symptomatic TB had a higher hazard of TB diagnosis than those without household TB exposure at baseline over the full study period, while the hazard of TB diagnosis among those exposed to asymptomatic TB was not significantly elevated. Hazard ratios for symptomatic household exposure were of a larger magnitude in sensitivity analyses restricted to two and three years of follow-up than over the full 11-year period, though these shorter-term estimates carried wider confidence intervals, likely reflecting the small number of events accrued over these narrower follow-up windows. This pattern suggests that household-associated TB risk may be concentrated in the period shortly after exposure, with some attenuation of this signal over longer follow-up as community-level transmission and unmeasured exposures accumulate. Individuals living in households with a participant with symptomatic TB had the highest cumulative probability of diagnosis over the follow-up period; however, confidence intervals were wide and overlapped substantially between groups due to the small number of events. This is consistent with meta-analytic evidence suggesting that less than 20% of community transmission is attributable to household exposure [29]. Importantly, individuals in the group unexposed to TB in their households at baseline also experienced a high cumulative probability of TB diagnosis, suggesting that exposure risk in this setting is not confined to identifiable household contacts but reflects widespread community transmission. These findings do not negate the increased transmission risk associated with household contacts nor the demonstrated effectiveness of household-based prevention strategies [30]. Rather, they highlight that while household exposure remains an important marker for targeted interventions, the relative contribution of symptomatic and asymptomatic individuals to community-level transmission requires more direct, prospective investigation, particularly in lower-burden settings. The cumulative probability of TB diagnosis among individuals living in households with symptomatic participants (13%) appears higher than estimates reported in household contact studies [31]. However, these are not directly comparable, as our estimates reflect cumulative risk over up to 11 years of follow-up and include TB diagnoses identified through routine health service data, capturing both recent transmission and disease arising from reactivation or subsequent exposures.

Several limitations warrant careful interpretation of these results. Incomplete linkage of ZAMSTAR participants to provincial health records may have introduced selection bias. Individuals with TB, HIV, or greater healthcare utilization may be more frequently captured within the PHDC and therefore are more likely to be successfully linked. Consequently, the linked cohort may not be fully representative of the original survey population. Although electronic data capture and integration into the PHDC have improved over time, further work is needed to strengthen patient identification systems to support robust longitudinal analyses such as this. People with TB are more likely to access public health care facilities and therefore be available for linkage in the PHDC database. As a result, absolute incidence estimates and some risk associations may be imprecise. Incomplete access to national vital registration data may have resulted in under-ascertainment of deaths occurring outside health facilities, with potential implications for time-to-event analyses. Participants without incident TB diagnosis or recorded death were assumed to contribute person-time until 31 December 2020. In reality, some individuals may have migrated out of the province, died without being recorded, or sought care outside the provincial health system. However, most participants (58%) had recorded contact with Western Cape health services after the study end date, suggesting that this assumption is unlikely to have substantially affected the findings. We cannot attribute incident TB definitively to household transmission over an 11-year period, as long time intervals and external exposures complicate causal inference. In addition, household exposure groups were defined at baseline and assumed to remain constant throughout follow-up, despite challenges in household composition and TB exposure over time, which may have resulted in exposure misclassification. Subgroup analyses within index households were limited by small numbers of incident events, reducing the statistical power to detect modest associations and to stratify by age, sex, or HIV. Sex and HIV status were included as established predictors of TB risk; however, we did not establish an empirical association between either variable and household exposure type and therefore cannot confirm that they act as true confounders of this association. Because hazard ratios from Cox models are non-collapsible, omitting a covariate strongly associated with the outcome can alter the estimated hazard ratio of another covariate even in the absence of confounding, which is why both were retained. The survey participants might also have been included in the ZAMSTAR interventions which could have influenced their subsequent behaviour and/or access to care. Finally, the survey reflects participants from eight Western Cape communities and is not fully representative of the province or country, limiting generalisability.

## Conclusions

This cohort offers long-term insights into TB risk in high-burden communities, including a higher hazard of TB diagnosis among those with household exposure to symptomatic TB, and highlights the value of unique patient IDs and linkage for understanding patterns of symptomatic and asymptomatic disease. Strengthening routine data systems, such as using unique patient IDs, and linkage processes, by ensuring rigorous procedures for demographic data collection, will further refine these estimates and improve our ability to detect important differences and support effective TB control in similar settings.

## Supporting information

S1 Table Terms and corresponding definitions. (DOCX)

S1 Text Description of the Provincial Health Data Centre deterministic linkage algorithm and criteria used to identify high-likelihood matches between ZAMSTAR participants and Western Cape health service records. (DOCX)

S1 Fig Comparison of demographic characteristics between participants in the ZAMSTAR survey who did and did not consent to sharing their information (top), and the consenters we could and could not link to the PHDC (bottom). (TIF)

S2 Table Annual TB incidence rate per 100,000 person-years between 2011 and 2020, numbers of individuals newly diagnosed per year and exposure times. (DOCX)

S2 Fig Kaplan-Meier survival curves of the probability of being diagnosed with TB by household type. Green, orange, and purple lines indicate baseline, asymptomatic, and symptomatic households respectively.
(TIF)

S3 Table Cox proportional hazards ratios of TB diagnosis risk by household type between 2010 and 2020 for the WHO symptom definition, N = 15,471. (DOCX)

S3 Fig Kaplan–Meier curves showing the cumulative probability of TB diagnosis over 2-year (A and C) and 3-year follow-up periods (B and D), stratified by household exposure type and symptom status (A and B: extended six symptoms, C and D: four WHO symptoms). Analyses were restricted to shorter follow-up windows to assess whether associations between household exposure and incident TB were concentrated in the early period following baseline. Individuals were censored at 2 and 3 years, respectively, if no TB diagnosis had occurred. Censoring lines representing death during the follow-up periods are not shown here for simplicity.
(TIF)

S4 Table Cox proportional hazard ratios of TB diagnosis risk by household type for follow-up periods of two and three years, split by symptom status. (DOCX)
