# Supplementary material for: Tuberculosis incidence among household contacts of individuals with symptomatic and asymptomatic tuberculosis: A historical cohort study in the Western Cape province of South Africa
Source: PLOS Glob Public Health. 2026 Oct 29;6(10):e0006550. doi: 10.1371/journal.pgph.0006550 (PMC13718926; doi:10.1371/journal.pgph.0006550)
Supplement: Supplementary file 1 [file pgph.0006550.s001.docx]

**S1 Table**: Terms and corresponding definitions

| **Term** | **Definition** |
| --- | --- |
| Incident TB | **ZAMSTAR**: An individual with a positive culture test for *Mycobacterium tuberculosis* from a respiratory sample.  **PHDC**: An individual with a positive laboratory test (including culture, smear microscopy, and/or molecular diagnostics such as Xpert MTB/RIF), or clinical diagnosis (ICD10 codes A15-A19, chest X-ray, symptom screening, history of TB contact, and/or clinical examination) if diagnosed after ZAMSTAR and included in the PHDC. |
| PLWH | **ZAMSTAR**: Positive test on Determine or Unigold based on serology for participants that produced blood for testing during ZAMSTAR, supplemented with self-reported status for those with no result from serology testing.  **PHDC**: HIV status after ZAMSTAR is based on routine HIV testing and ARV use. |
| Symptomatic TB during **ZAMSTAR** | Four WHO symptoms: presence of at least one symptom including cough, night sweats, fever, and weight loss. The ZAMSTAR trial assisted in deriving the 4SS tool. |
|  | Extended six symptoms: presence of at least one of the four WHO symptoms, extended to include chest pain and shortness of breath. |
| Asymptomatic TB during **ZAMSTAR** | Absence of symptoms under both categories. |

ZAMSTAR = Zambia, South Africa Tuberculosis and AIDS Reduction (ZAMSTAR) study, PHDC = Provincial Health Data Centre, PLWH = People living with HIV, WHO = World Health Organization
