# Supplementary material for: Tuberculosis incidence among household contacts of individuals with symptomatic and asymptomatic tuberculosis: A historical cohort study in the Western Cape province of South Africa
Source: PLOS Glob Public Health. 2026 Oct 29;6(10):e0006550. doi: 10.1371/journal.pgph.0006550 (PMC13718926; doi:10.1371/journal.pgph.0006550)
Supplement: Supplementary file 2 [file pgph.0006550.s002.docx]

**S1 Text:** Description of the Provincial Health Data Centre deterministic linkage algorithm and criteria used to identify high-likelihood matches between ZAMSTAR participants and Western Cape health service records.

The PHDC has developed a multi-step, rule-based, deterministic linkage algorithm coded in SQL and implemented in the Microsoft SQL Server environment^1^. The algorithm takes as input a variety of linkage fields (e.g., first name, surname, sex, date of birth, address). Whilst not all fields need be available in the input dataset, the power of the algorithm improves with the number of linkage fields provided. The 48 linkage rules act as progressive blocking criteria which are applied sequentially to identify and refine potential matches across datasets. A formal quantitative confidence score per match is currently not generated, but a set of six qualitative categories based on the details of the match acts as a proxy for confidence and is used to guide acceptance or review of matches. Only high-likelihood matches were accepted for this study.

1. Mutemaringa T, Heekes A, Smith M, Boulle A, Tiffin N. Record linkage for routinely collected health data in an African health information exchange. Int J Popul Data Sci. 2023;8(1):1771. https://doi.org/10.23889/ijpds.v8i1.1771
