# Supplementary material for: Tuberculosis incidence among household contacts of individuals with symptomatic and asymptomatic tuberculosis: A historical cohort study in the Western Cape province of South Africa
Source: PLOS Glob Public Health. 2026 Oct 29;6(10):e0006550. doi: 10.1371/journal.pgph.0006550 (PMC13718926; doi:10.1371/journal.pgph.0006550)
Supplement: Supplementary file 3 [file pgph.0006550.s004.docx]

**S2 Table:** Annual TB incidence rate per 100,000 person-years between 2011 and 2020, numbers of individuals newly diagnosed per year and exposure times

| **Year** | **Number of new diagnoses** | **Exposure time (person years)** | **Incidence rate (95% CI)** |
| --- | --- | --- | --- |
| 2011 | 251 | 15,548 | 1,614.33 (1,426.49 – 1,826.91) |
| 2012 | 237 | 15,562 | 1,522.92 (1,340.87 – 1,729.68) |
| 2013 | 203 | 15,596 | 1,301.6 (1,134.32 – 1,493.54) |
| 2014 | 206 | 15,593 | 1,321.09 (1,152.47 – 1,514.38) |
| 2015 | 208 | 15,591 | 1,334.09 (1,164.57 – 1,528.28) |
| 2016 | 185 | 15,614 | 1,184.82 (1,025.82 – 1,368.46) |
| 2017 | 191 | 15,608 | 1,223.72 (1,061.92 – 1,410.16) |
| 2018 | 197 | 15,602 | 1,262.64 (1,098.09 – 1,451.86) |
| 2019 | 203 | 15,596 | 1,301.6 (1,134.32 – 1,493.54) |
| 2020 | 111 | 15,688 | 707.54 (587.44 – 852.2) |
