# Supplementary material for: Tuberculosis incidence among household contacts of individuals with symptomatic and asymptomatic tuberculosis: A historical cohort study in the Western Cape province of South Africa
Source: PLOS Glob Public Health. 2026 Oct 29;6(10):e0006550. doi: 10.1371/journal.pgph.0006550 (PMC13718926; doi:10.1371/journal.pgph.0006550)
Supplement: Supplementary file 4 [file pgph.0006550.s006.docx]

**S3 Table:** Cox proportional hazards ratios of TB diagnosis risk by household type between 2010 and 2020 for the WHO symptom definition, N = 15,471

|  | **Total (%)** | **Incident TB** | **HR (95% CI)** | **aHR (95% CI)** |
| --- | --- | --- | --- | --- |
| **Household type**^‡^ | | | | |
| Background | 15,181 (98.1%) | 1,774 | Reference | Reference |
| Symptomatic | 168 (1.1%) | 31 | 1.72 (1.17 – 2.55) | 1.57 (1.12 – 2.19) |
| Asymptomatic | 122 (0.8%) | 14 | 1.19 (0.69 – 2.06) | 1.04 (0.62 – 1.74) |
