# Supplementary material for: Tuberculosis incidence among household contacts of individuals with symptomatic and asymptomatic tuberculosis: A historical cohort study in the Western Cape province of South Africa
Source: PLOS Glob Public Health. 2026 Oct 29;6(10):e0006550. doi: 10.1371/journal.pgph.0006550 (PMC13718926; doi:10.1371/journal.pgph.0006550)
Supplement: Supplementary file 5 [file pgph.0006550.s008.docx]

**S4 Table:** Cox proportional hazard ratios of TB diagnosis risk by household type for follow-up periods of two and three years, split by symptom status.

|  | **2- year follow-up period** | | **3- year follow-up period** | |
| --- | --- | --- | --- | --- |
|  | **Incident TB** | **HR (95% CI)** | **Incident TB** | **HR (95% CI)** |
| **Extended six symptoms** | | | | |
| Background | 494 | Reference | 627 | Reference |
| Symptomatic | 10 | 1.82 (0.97 – 3.41) | 12 | 1.73 (0.98 – 3.10) |
| Asymptomatic | 1 | 0.28 (0.04 – 1.96) | 2 | 0.43 (0.11 – 1.74) |
| **Four WHO symptoms** | | | | |
| Background | 494 | Reference | 627 | Reference |
| Symptomatic | 10 | 1.88 (1.00 – 3.51) | 12 | 1.78 (1.01 – 3.15) |
| Asymptomatic | 1 | 0.25 (0.035 – 1.78) | 2 | 0.39 (0.098 – 1.58) |
